# Human papillomavirus in prostate cancer: examining the evidence for a co-factor role

**DOI:** 10.3389/fmicb.2026.1836929

**Published:** 2026-06-05

**Authors:** Jingqi Zhang, Shuxin Li, Bo Yin

**Affiliations:** 1 Department of Urology, Shengjing Hospital, China Medical University, Shenyang, China; 2 Department of Urology, The First Hospital of Jilin University, Changchun, China

**Keywords:** carcinogenesis, human papillomavirus, molecular mechanisms, prostate cancer, tumor microenvironment

## Abstract

The etiological role of human papillomavirus (HPV) in Prostate cancer (PCa) remains unresolved due to stark contradictions between observational associations and molecular detection studies. Traditional models attempting to define HPV as an independent driver of PCa struggle to reconcile these contradictions. This review aims to critically evaluate the conflicting evidence and propose a new framework: HPV may primarily function as a molecular cofactor rather than an independent carcinogenic driver. Within this framework, we systematically explore how HPV infection, through its oncogenic proteins, induces genomic instability, fosters chronic inflammatory microenvironments, and interacts with other pathogens to act as a co-factor that synergistically promotes PCa initiation and progression in individuals with specific genetic backgrounds. This co-factor model not only reconciles existing contradictory data but also provides a novel framework for future research, emphasizing the need to identify HPV-associated PCa subtypes for precision prevention and therapy. We discuss the potential value and applicability of prevention strategies based on this co-factor model. Redefining HPV’s role in PCa is crucial for advancing precision risk stratification and targeted prevention.

## 1 Introduction

Human papillomavirus (HPV) is a small, double-stranded DNA virus belonging to the *Papillomaviridae* family, with over 200 genotypes currently identified (Wang et al., 2023; Khamisy-Farah et al., 2023; Shankar and Walker, 2025). Different HPV types exhibit distinct tissue tropism and pathogenic potential. They infect human skin and mucosal epithelial cells, leading to a spectrum of benign or malignant lesions (Wang et al., 2023; Brianti et al., 2017; Gautam et al., 2019). As one of the world’s most common sexually transmitted pathogens, HPV is primarily transmitted through sexual contact (Kiamba et al., 2025; Wang et al., 2025; Isaguliants et al., 2021). Common sites of infection include mucosal tissues such as the genital tract, anus, and oropharynx, with extremely high infection rates among sexually active adult men and women (Kiamba et al., 2025; Isaguliants et al., 2021; De Martino et al., 2025; Di Domenico et al., 2018). HPV infection is closely associated with the development of multiple cancers, particularly persistent high-risk HPV infection, which is a significant causative factor (Viens et al., 2016; Baba et al., 2025). HPV infection is a necessary condition for cervical cancer development, being implicated in over 99.7% of cervical cancer cases (Viens et al., 2016; Baba et al., 2025). In males, HPV is one of the primary causes of penile cancer, capable of inducing squamous cell carcinogenesis (Bansal et al., 2016; Wu et al., 2022). Furthermore, HPV infection is also closely linked to vulvar cancer and anal cancer (Bansal et al., 2016; Wu et al., 2022). Despite the well-established oncogenic role of HPV in these anogenital and oropharyngeal cancers, its etiological involvement in PCa remains one of the most contentious issues in uro-oncology.

PCa is the second most common malignant tumor among men globally, following lung cancer, and ranks as the third leading cause of cancer-related deaths in males (Al-Ghazawi et al., 2023; Nowacka-Zawisza and Wiśnik, 2017). It has become a significant public health issue worldwide. In recent years, the incidence and mortality rates of PCa have risen significantly, a trend most pronounced among elderly men (Pernar et al., 2018). HPV is a well-established carcinogenic pathogen. However, this causal relationship remains hotly debated in PCa. On one hand, some epidemiological studies and histological examinations suggest that HPV infection is associated with an increased risk of PCa, particularly in specific populations and with high-risk HPV genotypes (Tsydenova et al., 2023; Arriaga-Izabal et al., 2025). On the other hand, studies employing highly sensitive detection techniques often fail to consistently detect HPV nucleic acids in prostate tumor tissues (Lang et al., 2023; Andersen et al., 2024). This significant discrepancy in the evidence raises a critical scientific question. Is HPV a direct causative agent of PCa, a co-factor, or an unrelated factor? In conceptualizing HPV’s role, it is important to distinguish our proposed co-factor framework from existing models of viral carcinogenesis. The “hit-and-run” model posits that a virus initiates oncogenic transformation through transient genetic damage but is subsequently lost or no longer required for tumor maintenance (Lawson and Glenn, 2020). Conversely, the “driver vs. passenger” debate categorizes viral presence as either a causal, necessary factor (driver) or an incidental, non-functional infection (passenger). The co-factor model we propose diverges from these binary frameworks. It aligns more closely with the “hit-and-run” concept in acknowledging that HPV may not need to persist at high levels to exert its oncogenic influence. However, it extends this model by emphasizing a more sustained, synergistic role: rather than solely initiating transient damage, HPV lowers the oncogenic threshold and remodels the microenvironment in a manner that continues to facilitate tumor progression in concert with other genetic or environmental factors. This perspective is particularly relevant for the prostate, where intrinsic tissue biology may favor a context-dependent, synergistic role over a dominant driver function. This review will first critically evaluate the evidence supporting and opposing the association between HPV and PCa, delving into the underlying causes of this controversy. It will then systematically elucidate the potential molecular and immunological mechanisms through which HPV may contribute within this new framework. Finally, it will discuss the implications of this fresh perspective for PCa prevention and precision treatment strategies.

## 2 Methods

This review was conducted to critically evaluate the existing evidence on the association between HPV and PCa, with a specific focus on proposing a novel co-factor framework. A systematic literature search was conducted on PubMed and Web of Science. The search strategy combined keywords related to the virus (“Human papillomavirus,” “HPV”), the cancer (“PCa,” “PCa,” “prostatic neoplasm”), and their proposed relationship (“carcinogenesis,” “mechanism,” “epidemiology,” “etiology”). The search was limited to peer-reviewed articles published in English between January 1998 and May 2025 to capture both seminal and the most recent findings. We included original research articles (including case–control studies, cohort studies, and molecular mechanism studies), systematic reviews, and meta-analyses that investigated the presence, epidemiology, or molecular mechanisms of HPV in prostate tissue or in relation to PCa. We excluded case reports, conference abstracts, and non-English articles. The reference lists of included articles were also manually screened to identify additional relevant studies. The evidence was synthesized narratively to support re-evaluating HPV’s role from a traditional driver to a context-dependent co-factor. The results of this literature synthesis are presented below.

## 3 Observational evidence between HPV and PCa

Recent observational studies indicate that HPV infection rates among PCa patients differ significantly from those in healthy males, suggesting HPV may play a role in the development, progression, and treatment outcomes of PCa. However, divergent results and findings have been observed in other studies.

### 3.1 Observational evidence: HPV prevalence and risk associations

The presence of HPV in prostate tissue and its potential carcinogenic mechanisms have become a highly contentious field of research. Findings regarding HPV detection in prostate tissue exhibit marked heterogeneity across numerous studies. A study involving 162 patients with prostate disease revealed an overall HPV detection rate of merely 6.2% in prostate tissue (Pereira et al., 2023). Within the PCa cohort, the positivity rate was 7.4%, compared to 3.6% in the non-PCa group (Pereira et al., 2023). Although the detection rate was higher in the PCa group, the difference between groups did not reach statistical significance. A meta-analysis of 27 case–control studies, including 1,607 PCa tissue samples and 1,515 control samples (317 normal, 1,198 BPH), reported HPV detection rates of 25.8% in PCa patients, 9.2% in normal tissue, and 17.4% in BPH samples (Tsydenova et al., 2023). Moreover, a UK pilot study employing PCR and sequencing techniques analyzed 49 prostate specimens, reporting high-risk HPV (HR-HPV) presence in 32.7% of tissues, with significantly higher infection rates in abnormal tissue, although the small sample size limits the generalizability of this finding (Ahmed et al., 2023). However, studies employing next-generation sequencing (NGS) techniques failed to detect HPV RNA in malignant prostate tissue or adjacent normal tissue. HPV16 DNA was detected in only 0.8% of malignant samples, a result approaching the detection threshold (Andersen et al., 2024). In summary, although two studies suggest a potential association between HPV infection of the prostate and PCa, demonstrating temporal and spatial consistency and biological plausibility, particularly in specific populations and high-risk types, the current evidence remains highly heterogeneous. Further validation through larger-scale and prospective studies is required.

In recent years, the role of HPV in the development and progression of PCa has garnered increasing attention. A large-scale case–control study based on Taiwan’s National Health Insurance database enrolled 5,137 PCa patients and 15,411 cancer-free controls matched at a 1:3 ratio (Yin et al., 2024). The study found that the prevalence of HPV infection history was significantly higher in the PCa group than in the control group (Yin et al., 2024). The risk of developing PCa among HPV-infected individuals was 2.32 times higher than that of the control group (Yin et al., 2024). In a prospective study, researchers analyzed benign prostate biopsy samples from 52 Australian men who subsequently developed PCa to investigate the potential role and biological activity of high-risk HPV (Glenn et al., 2017). Results revealed detectable high-risk HPV in benign prostate tissue from patients who later developed PCa. Moreover, there was high concordance in HPV types between the benign and malignant tissues from the same patients (Glenn et al., 2017). TCGA data further demonstrated the presence of high-risk HPV16/18 transcripts in a minority of PCa samples, supporting the potential biological activity of HPV in certain tumors (Glenn et al., 2017). Moreover, a nested case–control study was conducted using a large Finnish male serum repository, involving 20,243 healthy men who were followed for up to 24 years (Dillner et al., 1998). By detecting IgG antibodies against four HPV types and Chlamydia in serum, the study found that HPV 18 antibody positivity was significantly associated with an increased risk of PCa (Dillner et al., 1998). HPV 16 infection also showed an elevated risk trend, whereas HPV 11, HPV 33, and Chlamydia infection demonstrated no significant association (Dillner et al., 1998). A study assessing HPV infection in PCa in an Indian population conducted PCR testing and genotyping on 95 PCa tissue samples and 55 BPH specimens (Singh et al., 2015). Results revealed an HPV infection rate of 41% in the PCa cohort, significantly higher than the 20% observed in the BPH group (Singh et al., 2015). Notably, the prevalence of HPV 16 infection reached 32% in the cancer group, demonstrating a highly significant difference compared to the control group (Singh et al., 2015). A systematic review employing Bradford Hill criteria to assess the causal relationship between HPV and PCa analyzed 60 relevant studies (Opeyemi Bello et al., 2023). The findings indicate a weak association between the two, insufficient to support HPV as a causative factor for PCa (Opeyemi Bello et al., 2023). The observational association between HPV infection and PCa exhibits substantial discordance, primarily stemming from methodological variations, sample heterogeneity, and limitations in detection techniques. While some studies support HPV as a co-factor or synergistic risk factor for PCa, the overall quality of evidence remains low.

### 3.2 Current challenges in the association between HPV and PCa

HPV is a highly carcinogenic virus closely associated with the development of multiple malignant tumors, though its link to PCa remains a subject of debate to this day (Yin et al., 2024). A study suggested that HPV infection may increase PCa risk. For instance, a Mexican cohort study reported an odds ratio of 2.34 for PCa in HPV-positive samples, with 77% of positive samples being high-risk HPV types (Arriaga-Izabal et al., 2025). Nevertheless, two studies have failed to confirm this association (Andersen et al., 2024; Nellessen et al., 2023). Indeed, systematic reviews have indicated that most effect estimates do not support a causal relationship between HPV and PCa (Opeyemi Bello et al., 2023; Noorani et al., 2025). This inconsistency in findings results in low overall evidence quality. Although biological plausibility is supported to some extent, causality remains far from established. Current studies predominantly employ retrospective designs, lacking prospective cohort evidence. They are susceptible to selection and recall biases and often fail to adequately incorporate clinical risk factors. Furthermore, variable HPV detection methods significantly impair the comparability of results, with some studies relying solely on single techniques rather than multiplex detection strategies, leading to substantial variations in detection rates (Tsydenova et al., 2023; Andersen et al., 2024). This explains why different research teams arrive at divergent conclusions. For instance, a meta-analysis based on NGS technology found no evidence that HPV is a primary cause of PCa, whereas other studies using tissue-based detection reported positive associations (Andersen et al., 2024). And limitations in detection technique sensitivity may yield false-negative or false-positive results. For instance, one study confirmed HPV E7 protein expression in individual samples via immunohistochemistry, yet reported an extremely low overall HPV DNA detection rate, further highlighting the impact of methodological differences on conclusions (Tsydenova et al., 2023; Ahmed et al., 2023). Geographical variations also fuel this debate. For example, while HPV infection significantly increases PCa risk in Mexican men, a study in a Chinese population found no significant association between HPV status and genomic alterations in PCa (Arriaga-Izabal et al., 2025; Lang et al., 2023). These conflicting findings suggest that the positive association observed in Mexican men may not be generalizable to other populations. Inappropriate control group selection may also weaken the strength of the association. For instance, some studies included controls with pre-existing HPV infection, which could dilute risk estimates (Tsydenova et al., 2023; Arriaga-Izabal et al., 2025). Inadequate control of confounding variables similarly generates conflicting effect estimates. For example, a Taiwanese study reported that prostatitis or a history of sexually transmitted infections increased HPV-associated PCa risk; however, only a limited number of studies have adequately adjusted for such factors (Yin et al., 2024). Further studies with more rigorous control of confounders are needed. Furthermore, variations in HPV genotype distribution contribute to these findings. The predominance of high-risk types may amplify risk associations, whereas the presence of low-risk types may attenuate the overall effect (Arriaga-Izabal et al., 2025).

Beyond methodological and epidemiological variables, the unique biological microenvironment of the prostate may fundamentally shape the interaction with HPV and influence its detectability. Unlike the transformation zones of the cervix, which are highly permissive to HPV replication and persistence, the prostate epithelium presents a distinct milieu (Singh et al., 2021; Li et al., 2020). High physiological concentrations of zinc, which possess antimicrobial properties, may inherently suppress viral replication or promote a latent state, making HPV more difficult to detect using standard nucleic acid assays (Li et al., 2020; Mullin et al., 2017; Shafiei et al., 2025). Furthermore, the prostate exhibits relative immunological quiescence compared to mucosal sites, potentially leading to a less robust inflammatory response to HPV infection and facilitating viral evasion (Emtiazi et al., 2025). It is important to note that the following considerations are speculative and require experimental validation. These intrinsic tissue-specific factors could hypothetically predispose HPV to act not as a dominant, persistent driver, but as an intermittent or low-level co-factor. In this scenario, HPV’s oncogenic proteins might conjecturally exert their effects episodically or in a ‘hit-and-run’ manner, leaving a fainter molecular footprint that is easily missed in cross-sectional studies focused on viral DNA presence. This biological perspective, while hypothetical, aligns with the co-factor model, suggesting that HPV’s role in the prostate may be context-dependent and modulated by the local tissue ecology, which could offer an explanation for why its signature is elusive and inconsistent across studies.

Beyond these methodological considerations, residual confounding remains a fundamental challenge in interpreting the HPV-PCa association. Sexual behavior, a primary determinant of HPV exposure, is also independently associated with PCa risk through pathways unrelated to HPV (e.g., hormonal influences, chronic inflammation from other sexually transmitted infections) (Noorani et al., 2025; Caini et al., 2014; Cherven et al., 2021). Insufficient adjustment for sexual history is a well-known variable that is difficult to measure accurately. Therefore, false associations may arise. Similarly, co-infection with other sexually transmitted pathogens (e.g., *Chlamydia trachomatis*, Trichomonas vaginalis, or EBV) may independently contribute to prostatic inflammation and carcinogenesis, potentially confounding estimates of HPV’s specific contribution (Pei et al., 2025; Nahand et al., 2021; Ghosh et al., 2017). Socioeconomic factors further complicate this landscape speculatively, as they may influence both HPV acquisition (through access to healthcare, health literacy, and sexual networks) and PCa detection (through disparities in screening access and health-seeking behaviors), although direct evidence in the context of HPV-PCa remains limited (Coughlin, 2020; Järbur et al., 2025). The interplay between these factors creates a complex web of potential biases that few studies have adequately addressed, underscoring the need for more sophisticated analytical approaches in future investigations.

## 4 Potential mechanisms of HPV in the development and progression of PCa

Despite the heterogeneity of epidemiological evidence and the possibility that HPV may not be an independent driver of PCa development, mounting molecular evidence supports HPV as a significant co-factor in the pathogenesis of PCa. Critically, the mechanisms elucidated below are consistent with a role wherein HPV lowers the oncogenic threshold and creates a permissive landscape for tumorigenesis, rather than acting as a sole initiator. The direct actions of viral oncoproteins and the indirect remodeling of the tumor microenvironment collectively illustrate how HPV can amplify existing risks and synergize with other factors.

### 4.1 Direct effects of HPV oncogenic proteins

**Figure 1:**
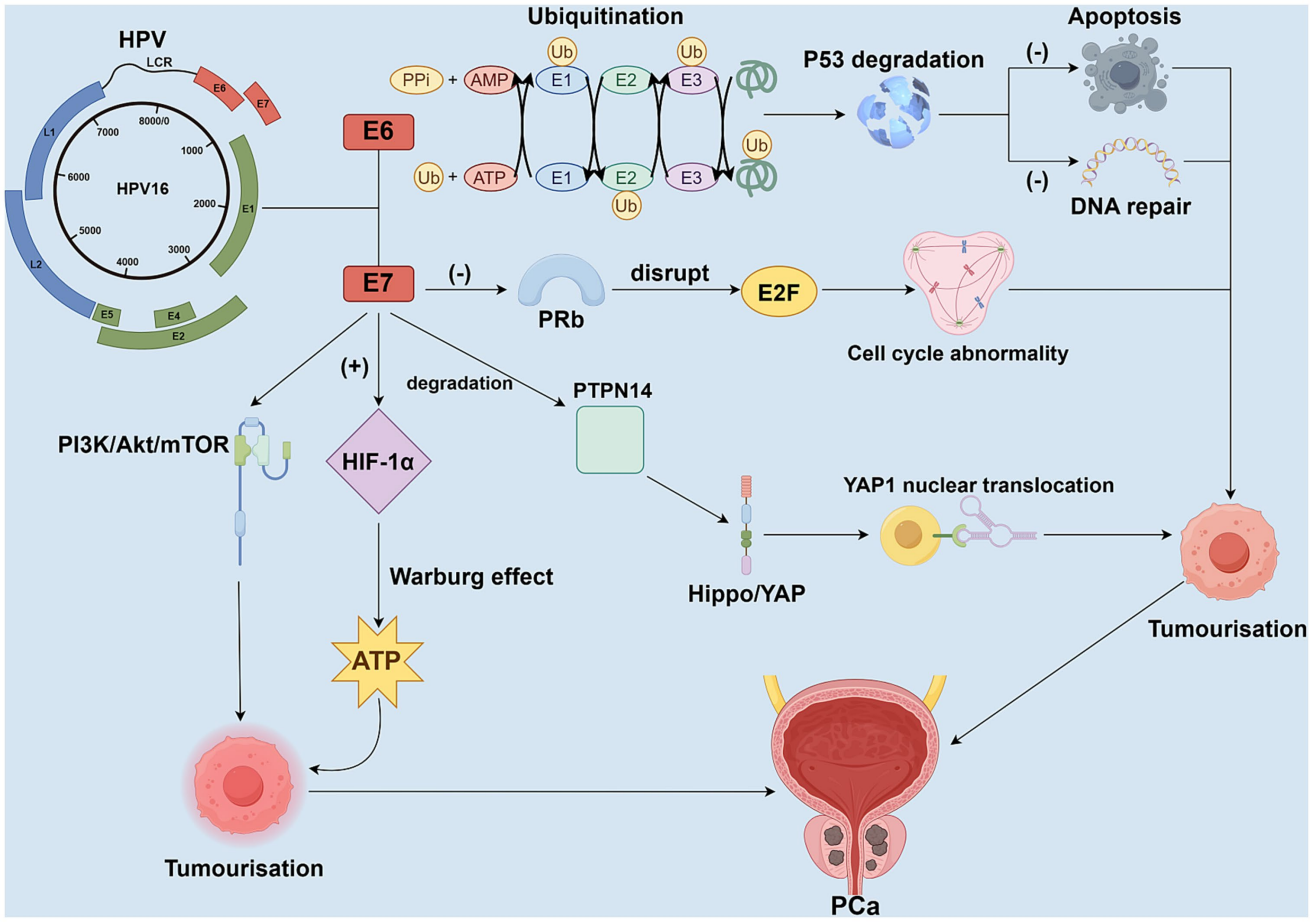
Proposed direct molecular mechanisms underpinning the co-factor role of HPV in PCa. These mechanisms are largely extrapolated from well-established findings in other HPV-driven cancers (e.g., cervical cancer) and remain speculative in the prostate context until further validation. The E6 and E7 oncoproteins of high-risk HPV types are proposed to contribute to prostate carcinogenesis by targeting key cellular regulators, thereby creating a permissive state for transformation. E6 promotes the degradation of tumor suppressor p53, impairing apoptosis and DNA repair. E7 inactivates retinoblastoma protein (pRb), leading to uncontrolled cell cycle progression. Additionally, E6/E7 may activate oncogenic signaling pathways such as PI3K/Akt/mTOR and Hippo/YAP, induce metabolic reprogramming through HIF-1α stabilization, and enhance cell survival and proliferation.

The direct molecular interventions of HPV provide a plausible mechanism for its cofactor activity. However, it is important to note that much of our understanding of HPV oncoprotein functions derives from well-characterized HPV-driven cancers, particularly cervical cancer. While these mechanisms offer a biologically plausible framework, their direct applicability to prostate carcinogenesis requires further validation. With this caveat, the evidence suggests that HPV oncoproteins may deregulate pre-existing cellular safeguards, thereby lowering the barrier to malignant transformation. Specifically, these proteins may contribute to the process through multiple mechanisms, as summarized in Figure 1. Evidence directly demonstrated in prostate tissue includes the following: Studies indicate that HPV E7 protein expression levels are significantly higher in benign prostate tissue than in advanced PCa tissue, suggesting that its oncogenic activity may primarily influence early-stage PCa development (Glenn et al., 2017). Furthermore, immunohistochemical analysis confirmed the presence of HPV E7 protein expression in prostate tissue and revealed an association between HPV E7 expression and PCa progression (Ahmed et al., 2023). Additionally, persistent overexpression of p16-INK4A protein, a well-established surrogate marker of HPV E7 oncogenic activity, was consistently observed in HPV-positive prostate samples, further substantiating the potential involvement of HPV infection in PCa development (Medel-Flores et al., 2018; Kwon et al., 2019). From studies in cervical and head and neck cancers, the E6 and E7 proteins may promote genomic instability and mutation accumulation in host cells by interfering with DNA damage repair pathways, potentially laying the genetic foundation for malignant transformation (Obanya et al., 2025). Specifically, research in cervical cancer models has shown that the E6 protein is known to mediate the ubiquitin-mediated degradation of the tumor suppressor protein p53, thereby potentially inhibiting apoptosis and impairing DNA repair capacity (Obanya et al., 2025; Paolini et al., 2021; Đukić et al., 2020). Meanwhile, evidence from HPV-transformed cell lines indicates that the E7 protein has been shown to bind to and inhibit the retinoblastoma protein (pRb), thereby potentially releasing its regulatory control over the transcription factor E2F (Obanya et al., 2025; Paolini et al., 2021; Đukić et al., 2020). This may contribute to abnormal progression of the cell cycle and uncontrolled proliferation. Studies in HPV-associated cancers have further revealed that HPV oncoproteins E6/E7 are not only capable of suppressing tumor suppressors like p53 and pRb but also may activate the PI3K/Akt/mTOR signaling pathway (Zhang et al., 2015). This pathway is involved in regulating cellular processes, including proliferation, survival, and metabolism, thereby potentially promoting virus-mediated cellular immortalization and tumor progression (Zhang et al., 2015). Additionally, findings from cervical cancer research demonstrate that the E7 protein has been reported to modulate the Hippo/YAP signaling pathway by degrading the tumor suppressor protein PTPN14, thereby potentially promoting the nuclear translocation of the oncogenic protein YAP1 (Blakely et al., 2024). This mechanism may not only contribute to maintaining epithelial cell stemness and self-renewal capacity but also could accelerate tumor progression (Coughlin, 2020). Studies in cervical and other HPV-driven cancers have also shown that E6/E7 proteins have also been implicated in enhancing the stability of HIF-1α, a key regulator of glycolysis, thereby potentially triggering the Warburg effect (Gore et al., 2025; Arizmendi-Izazaga et al., 2024). This metabolic shift may fuel the rapid proliferation of cancer cells and contribute to creating a microenvironment conducive to tumor progression (Gore et al., 2025; Arizmendi-Izazaga et al., 2024). In summary, while the direct demonstration of these mechanistic pathways in prostate tissue remains limited, the existing evidence from other HPV-associated cancers provides a biologically plausible framework for understanding how HPV oncoproteins may contribute to PCa development. Further studies are needed to validate whether these mechanisms operate similarly within the unique prostate microenvironment.

### 4.2 Indirect carcinogenic mechanism of HPV infection

**Figure 2:**
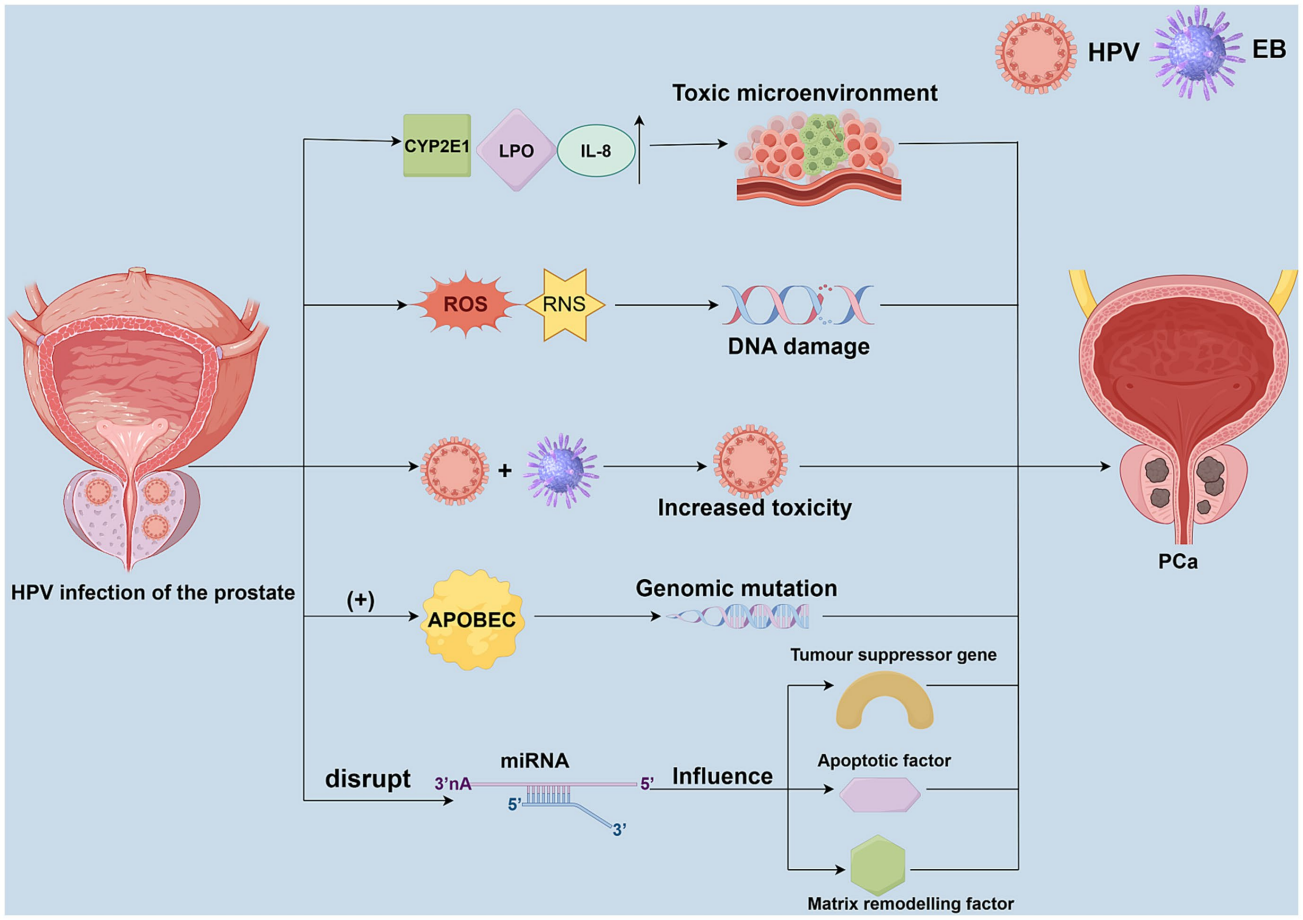
Hypothetical indirect microenvironmental mechanisms supporting the HPV co-factor hypothesis. These mechanisms are partly extrapolated from other HPV-associated cancers and partly speculative, requiring direct evidence in prostate tissue. HPV infection is speculated to contribute to prostate carcinogenesis by remodeling the local tissue environment. This includes establishing a chronic inflammatory state characterized by elevated oxidative stress and pro-inflammatory cytokines, which may cause DNA damage and suppress local immunity. HPV may also synergize with other pathogens, trigger host APOBEC-mediated mutagenesis, and dysregulate miRNA expression profiles. These alterations could collectively foster a niche conducive to tumor initiation, immune evasion, and progression.

Beyond direct cellular manipulation, HPV may foster prostate carcinogenesis indirectly by reshaping the tissue microenvironment into a pro-tumorigenic niche. This indirect role as a co-factor involves establishing a chronic inflammatory state and altering local immune dynamics, which can promote the survival and expansion of initiated cells regardless of the initiating cause. Evidence for these indirect mechanisms derives from a combination of prostate-specific studies and extrapolations from other HPV-associated cancers; where possible, we distinguish the source of evidence. Prostate-specific evidence indicates that high-risk HPV infection may trigger such a remodeling of the epithelial microenvironment, potentially establishing a chronic inflammatory state (Figure 2). This persistent inflammation may promote viral persistence and could accelerate disease progression by altering the local immune microenvironment (Chaberek et al., 2022). Specifically, HPV infection has been associated with elevated oxidative stress markers (CYP2E1, LPO) and proinflammatory factors (IL-8), thereby potentially creating a toxic microenvironment (Pérez-Soto et al., 2022). The sustained release of inflammatory cytokines may contribute to DNA damage and uncontrolled cell proliferation, thereby creating favorable conditions for carcinogenesis. Chronic inflammation can also indirectly contribute to tumorigenesis by inducing DNA damage through reactive oxygen species (ROS) and reactive nitrogen species (RNS) (Pereira et al., 2023; Javier-DesLoges et al., 2022). Furthermore, the HPV-induced inflammatory microenvironment may contribute to local immune suppression, potentially weakening antitumor immune responses, enabling infected cells to evade immune surveillance, and facilitating tumor growth (Shamseddine et al., 2021; Sfanos et al., 2018). HPV may also synergize with other microorganisms to potentially promote PCa progression (Nahand et al., 2021; Ghosh et al., 2017). Both HPV and EBV have been detected to coexist in normal, benign, and malignant prostate tissues, with EBV potentially enhancing HPV’s carcinogenic potential (Nahand et al., 2021). Conversely, HPV may indirectly contribute to carcinogenesis by activating host APOBEC (apolipoprotein B mRNA editing enzyme) proteins (Lawson and Glenn, 2020; Chen et al., 2017). APOBECs are known to mediate extensive genomic mutations during antiviral defense, potentially causing collateral damage to key tumor suppressor genes, which may represent a distinct HPV-mediated pathway in PCa progression (Lawson and Glenn, 2020; Chen et al., 2017). High-risk HPV infection has been associated with disrupted miRNA expression profiles that may promote tumorigenesis (Salgado-Hernández et al., 2024; Khatami et al., 2022). HPV-positive cancer tissues have been reported to exhibit distinct miRNA expression patterns, including significant upregulation of miR-19a and miR-21, alongside downregulation of miR-23b and miR-34a (Salgado-Hernández et al., 2024; Khatami et al., 2022). These miRNAs are thought to participate in regulating multiple malignant biological processes, including tumor cell proliferation, apoptosis, invasion, and immune response, by targeting tumor suppressor genes, apoptosis-related factors, and matrix remodeling molecules (Khatami et al., 2022). In summary, HPV infection may contribute to PCa initiation and progression through indirect mechanisms, including chronic inflammation, dysregulation of gene expression, APOBEC mutagenesis, and multipathing synergy.

## 5 Discussion

This review critically evaluates the conflicting evidence regarding the association between HPV and PCa. The traditional binary model of causality, which attempts to frame HPV as either a present or absent independent driver of PCa, struggles to reconcile the stark contradictions between observational findings and molecular detection studies. We propose a co-factor model that posits HPV as a context-dependent risk amplifier that synergizes with other factors under specific conditions, rather than a ubiquitous independent carcinogen.

The novelty of our contribution lies in its prostate-specific contextualization. We integrate the unique biological features of the prostate — including its high zinc concentration, immunological quiescence, and susceptibility to chronic inflammation — into a cohesive framework that explains why HPV’s oncogenic footprint is elusive and inconsistent. This organ-specific integration reconciles long-standing contradictions in the field.

A caveat concerns the source of mechanistic evidence. The molecular pathways involving E6/E7 are well-established in cervical and other HPV-associated cancers, but direct evidence for these mechanisms operating within the prostate microenvironment remains limited. Much of our mechanistic framework is extrapolated, and definitive demonstration awaits future investigation.

In addition to the postulated co-factor role of HPV E6/E7 oncoproteins in prostate carcinogenesis, a broader understanding of HPV’s biology in cancer includes the immune-based mechanisms of HPV-targeted prevention and the prognostic implications of HPV positivity. The licensed preventive HPV vaccines are primarily composed of the major capsid protein L1, which self-assembles into virus-like particles (VLPs) (Huber et al., 2021). These VLPs are highly immunogenic and elicit robust neutralizing antibody responses that block initial HPV infection at the basement membrane, thereby preventing viral entry and subsequent establishment of persistent infection (Barra et al., 2019). This L1-based prophylactic mechanism is fundamentally distinct from the direct oncoprotein-driven or co-factor activities of E6/E7. If a subset of prostate cancers is indeed associated with HPV infection, then L1 VLP vaccination could theoretically reduce the incidence of such cases by preventing initial acquisition of the virus (Pereira et al., 2023; Simons et al., 2020). However, this remains speculative, and current evidence does not support population-level vaccination specifically for prostate cancer prevention.

Furthermore, accumulating evidence from other HPV-associated malignancies, such as oropharyngeal squamous cell carcinoma, indicates that HPV-positive tumors often exhibit a better clinical prognosis compared to their HPV-negative counterparts (Welters et al., 2018). This favorable outcome is thought to be driven by a distinct tumor immune microenvironment, including enhanced immune cell infiltration (e.g., CD8 + T cells) and a higher susceptibility to immune-mediated elimination (Welters et al., 2018; Li et al., 2026). Whether this phenomenon extends to a potential HPV-associated subset of prostate cancer is entirely unknown. If confirmed, it would have profound implications for risk stratification and immunotherapy decision-making in prostate cancer. Therefore, future studies should not only focus on detecting HPV DNA or proteins in prostate tissues but also characterize the immune landscape and clinical trajectory of putative HPV-positive prostate cancer cases. Such investigations would help determine whether HPV status in the prostate serves as a prognostic biomarker or merely an incidental finding.

If an HPV-associated PCa subtype is definitively identified, preventive vaccination and HPV-directed therapies could theoretically reduce disease burden in that subset. However, given the current weak and discordant evidence, the broad application of these interventions is not justified. Future research must first define and validate HPV-associated PCa subtypes through multi-omics approaches before any clinical translation of HPV-targeted prevention or treatment can be considered. Cost-effectiveness analyses should await empirical data on the prevalence and clinical impact of such a subtype.

In conclusion, while the co-factor model reconciles many contradictions regarding HPV’s role in prostate cancer initiation and progression, it must be considered alongside the well-established L1-based prevention paradigm and emerging evidence on HPV-associated cancer immunobiology. Redefining HPV’s role in prostate cancer is not only crucial for mechanistic understanding but also for opening future avenues in precision prevention, risk stratification, and immunotherapy.
